# Effect of Dielectric Properties of a Solvent-Water Mixture Used in Microwave-Assisted Extraction of Antioxidants from Potato Peels

**DOI:** 10.3390/antiox3010099

**Published:** 2014-02-24

**Authors:** Ashutosh Singh, Gopu Raveendran Nair, Pansa Liplap, Yvan Gariepy, Valerie Orsat, Vijaya Raghavan

**Affiliations:** Department of Bioresource Engineering, McGill University, 21111, Rue Lakeshore, Ste-Anne-de-Bellevue, Quebec H9X 3V9, Canada; E-Mails: gopu.nair@mail.mcgill.ca (G.R.N.); pansa.liplap@mail.mcgill.ca (P.L.); yvan.gariepy@mcgill.ca (Y.G.); valerie.orsat@mcgill.ca (V.O.); vijaya.raghavan@mcgill.ca (V.R.)

**Keywords:** dielectric properties, microwave-assisted extraction, dielectric constant, dielectric loss, extraction solvent, plant matrix, phenolics

## Abstract

The dielectric properties of a methanol-water mixture were measured at different temperatures from 20 to 80 °C at two frequencies 915 MHz and 2450 MHz. These frequencies are most commonly used on industrial and domestic scales respectively. In this study, the dielectric properties of a methanol-water mixture were found to be dependent on temperature, solvent concentration, and presence of plant matrix. Linear and quadratic equations were developed to establish the dependency between factors. At 2450 MHz, the dielectric constant of methanol-water mixtures was significantly affected by concentration of methanol rather than by temperature, whereas the dielectric loss factor was significantly affected by temperature rather than by methanol concentration. Introduction of potato peel led to an increase in the effect of temperature on the dielectric properties of the methanol fractions. At 915 MHz, both the dielectric properties were significantly affected by the increase in temperature and solvent concentration, while the presence of potato peel had no significant effect on the dielectric properties. Statistical analysis of the dissipation factor at 915 and 2450 MHz revealed that both temperature and solvent concentration had a significant effect on it, whereas introduction of potato peels at 915 MHz reduced the effect of temperature as compared to 2450 MHz. The total phenolic yield of the microwave-assisted extraction process was significantly affected by the solvent concentration, the dissipation factor of the methanol-water mixture and the extraction time.

## 1. Introduction

Potato (*Solanum tuberosum* L.) plays an important role in human nutrition. It is considered as a good source of carbohydrates, fiber, and vitamins as well as of several beneficial phytonutrients like polyphenols and carotenoids. Conventionally these phytonutrients are extracted using heat-reflux or Soxhlet-extraction methods [1,2]. These methods have several disadvantages including, long extraction time, requirement of a high volume of samples and solvents and the overall yield is low [2]. In recent years the microwave-assisted extraction (MAE) process has gained considerable attention as an alternative to conventional methods of extraction [2]. It is a novel process, which utilizes microwave energy to increase the mass transfer of the analyte from the biological matrix into the solvent by volumetrically heating the solvent and the matrix [3]. When a microwave passes through a biological medium, its energy is absorbed and converted into thermal energy. The ability of the medium to absorb and convert microwave energy into heat is governed by its dielectric properties. The dielectric properties of the medium can be defined by the complex relative permittivity ε* (relative to that of free space) in the relationship ε* = ε′* − j*ε″*,* where *j* = √(−1), ε′, is the dielectric constant, which governs the ability of the medium to absorb microwave energy and ε″, the dielectric loss factor that is concerned with the conversion of absorbed microwave energy to heat. The relation between ε′ and ε″ is presented by the tangent of loss angle (tanδ = ε′/ε″), which along with dielectric constant defines the attenuation of microwave power within a biological matrix [4,5]. The dielectric properties of a solvent are one of the primary features for its selection as the extracting solvent in the MAE process. Several researchers have studied the effect of dielectric properties of solvents on the overall efficiency of the MAE process [6,7,8]. The general consensus states that the dielectric properties of the solvent used in the extraction process determine its ability to extract nutrients from a selected plant matrix. However, it is interesting to note that the dielectric properties of the solvent change when a biological matrix is added to it or if the extraction conditions are changed. Several studies have provided insight on the effect of temperature and microwave frequency on dielectric properties of binary mixtures of two different alcohols and alcohols with water [6,7,8].

In the present study we describe how the dielectric properties of a methanol-water mixture vary with changes in temperature, methanol concentration and presence of sample matrix (potato peel) at microwave frequencies of 2450 MHz and 915 MHz.

## 2. Experimental Section

### 2.1. Materials

Potato samples (cv. “Russet Burbank”) were obtained from the Elite Potato Centre at Bon Accord (NB, Canada). Tubers were washed and peeled with a mechanical peeler to obtain uniformity in thickness of the peel. The peel sample (approximately 100 g) was lyophilized in a laboratory freeze-dryer (Thermo Savant Modulyod-115, NY, USA) until a constant weight was obtained. After drying, the samples were ground to pass a standard 150 µm sieve, thus insuring uniformity and symmetry of particle size. The freeze-dried powder was kept in closed opaque containers at −20 °C until analysis. All reagents and solvents used were of HPLC grade (Fisher Scientific, Ottawa, ON, Canada). Double distilled water was used to prepare the solvent-water mixtures for analysis.

### 2.2. Equipment and Apparatus

**Figure 1:**
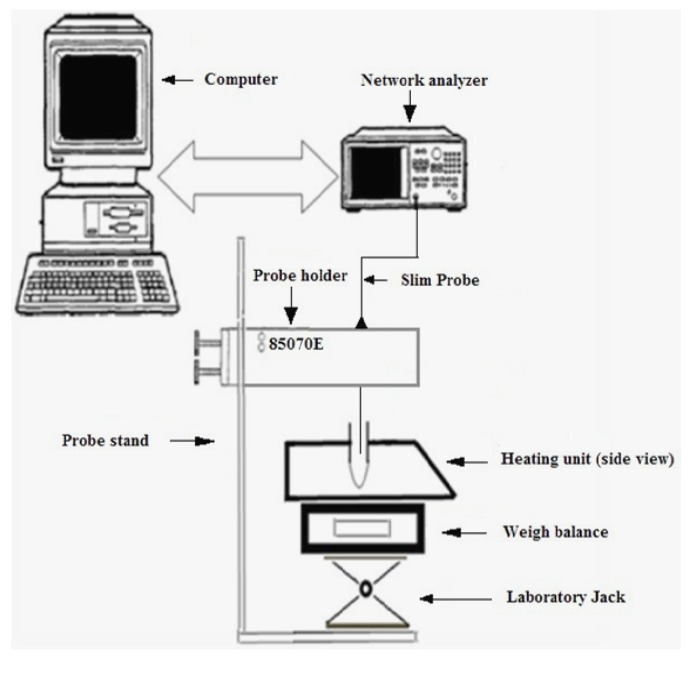
Schematic of the experimental setup for measurement of dielectric properties of methanol fractions (Source: Adapted from [9]).

An open-ended coaxial 2 mm diameter slim probe (85070E) and an automated Agilent 8722 ES S-parameter Network Analyzer (Santa Clara, USA) were used to determine the dielectric properties of the samples (Figure 1). Three point calibration of the system was performed with air, short block and water as described in the manual of Agilent Technologies (2001). The sample to be measured was taken in a small cylindrical test tube (20 mm in diameter, 50 mm height and 2 mm thickness) made of borosilicate glass. The dielectric properties (ε′ and ε″) were measured from 20 to 80 °C at 20 °C intervals for the frequencies 2450 and 915 MHz. Three repetitive measurements were performed at each temperature for each sample of the solvent-water mixture with peel and without peel. The probe was cleaned after every measurement.

### 2.3. MAE Extraction

MAE extraction was carried out with a focused open-vessel microwave system (Star System 2, CEM Matthews, USA) operating at a maximum power of 800 W and at a frequency of 2.45 GHz. The mode of microwave power applied was intermittent with power on for 30 s·min^−1^ [2].

### 2.4. Preparation of Potato Peel Extracts

The phenolic compounds were extracted from freeze-dried potato peels using 30, 65 or 100% (v/v) methanol (MeOH). Methanol was selected as the extraction solvent because it yielded the highest total phenolic content compared to ethanol during initial experiments. Solvent (40 mL) was added to 2 g of dried potato peel in a Pyrex vessel and placed inside the microwave, where phenolics were extracted at different power levels (10, 20 and 30% W) over different periods of time (5, 10 and 15 min). The extract was allowed to cool at room temperature and was then centrifuged at 10,000 rpm for 15 min. The supernatant was collected and used for total phenolics determination [2].

### 2.5. Determination of Total Phenolic Compounds

Total soluble phenolic content was determined using Folin-Ciocalteu reagent [2]. Potato peel extract solution (1 mL) was mixed with 7.5 mL of double distilled water and 0.5 mL of Folin-Ciocalteu reagent followed by 1 mL of 5% Na_2_CO_3_ solution. The mixture was incubated at room temperature for 90 min and its absorbance was measured at 765 nm using a spectrophotometer (Ultrospec 2100 pro, Biochrom Ltd., Cambridge, England). A standard curve was plotted using different concentrations of gallic acid and the amount of total phenolic content was expressed in terms of gallic acid equivalents (GAE) in mg per 100 g of potato peel (dry weight).

### 2.6. Statistical Analysis

Statistical analysis was carried out using SAS^®^ (version 9.2, SAS Institute, Cary, NC, USA) to determine the interaction between solvent concentration and temperature and their effect on dielectric constant, loss factor and loss tangent. Effect of dielectric constant, dielectric loss dissipation factor (tanδ) and extraction time on total phenolic content obtained from MAE extract was also analyzed.

## 3. Results and Discussion

### 3.1. Effect of Temperature on Dielectric Properties of Methanol-Water Mixtures

Organic solvent-water mixtures exhibit different dielectric properties at microwave frequencies; this property depends on their polarity, dipole strength and composition [8]. In this study we analyzed the dielectric properties of a methanol-water mixture with and without plant matrix in it at different temperatures and with different methanol volume fractions at 2450 and 915 MHz.

**Figure 2:**
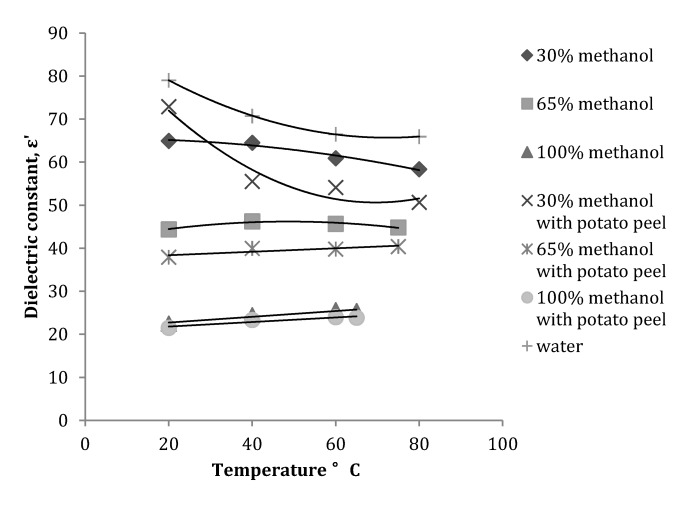
Variation in dielectric constant of a methanol-water mixture with and without potato peel at 2450 MHz (Source: Adapted from [9]).

It was observed that at 2450 MHz the dielectric constant for 30% methanol fraction (Figure 2) decreased with an increase in the temperature, whereas for 65% and 100% methanol fractions the value for the dielectric constant increased with an increase in the temperature. It was interesting to note that when plant matrix (potato peel) was introduced in the solvent with a solid to liquid ratio of 1:20, the dielectric constant for the 30% methanol fraction was greater at 20 °C compared to one without potato peel; with increase in temperature the values decreased and were lower than the values measured for the 30% methanol fraction without potato peel. For the 65 and 100% methanol fractions with potato peel the dielectric constant showed a gradual increase with an increase in the temperature and values measured were lower than values for methanol fractions without potato peel. The reason for the decrease and increase in dielectric constant values can be attributed to the change in the composition of the solvent-water mixture with the presence of the plant matrix [8,10]. There is no clear explanation for the changes in the behavior of the 30% methanol fraction; addition of plant matrix should basically add more polarity to the solvent mixture and this should increase the dielectric constant of the solvent-plant matrix mixture.

**Figure 3:**
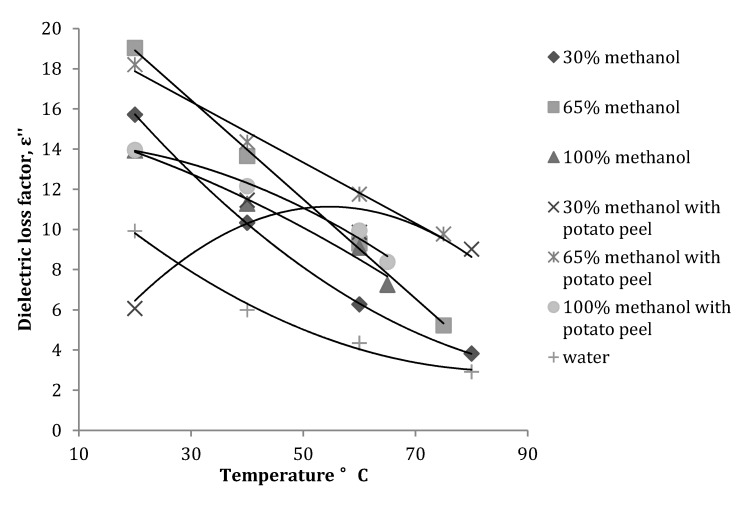
Variation in dielectric loss factor of a methanol-water mixture with and without potato peel at 2450 MHz (Source: Adapted from [9]).

At 2450 MHz, the dielectric loss factor decreased with an increase in temperature (Figure 3) for all the methanol fractions. Introduction of potato peel in the solvent mixture considerably reduced the rate of decrease in dielectric loss factor and the values measured at different temperatures were higher than the values obtained for methanol fractions without potato peel. This is explained by the fact that added plant material also absorbs microwave energy, which is later dissipated as heat. For the 30% methanol fraction with potato peel, the dielectric loss factor increased with an increase in temperature until 40 °C and then gradually decreased with further rise in temperature.

Statistical analysis of the data obtained for dielectric constant and dielectric loss factor at 2450 MHz with or without potato peel revealed that the dielectric constant of the methanol fractions was significantly (*p* ≤ 0.05) affected by the concentration of methanol rather than temperature (*p* ≥ 0.05). Conversely, the dielectric loss factor was more significantly (*p* ≤ 0.05) affected by the temperature of the process than the concentration (*p* ≥ 0.05) of methanol. When potato peels, were introduced in the methanol-water mixture, the dielectric constant was affected both by temperature (*p* ≤ 0.05) and concentration of the solvent (*p* ≤ 0.05), while the dielectric loss factor was only affected by temperature (*p* ≤ 0.05) and concentration played a minimal role in the change of dielectric loss. Regression analysis was performed to relate the effect of the methanol fraction concentration and temperature on ε′ and ε″ (Table 1).

**Table 1:** Equations defining the relationship between ε′ and ε″ with temperature and methanol fraction concentration at 2450 MHz (Source: Adapted from [9]).

Dielectric Properties	Plant material	Frequency (MHz)	Equation obtained	*R* ^2^
ε′	Potato peel	2450	78.65 − 0.51 Met − 0.11 Temp	0.91
ε″	Potato peel	2450	12.81 − 0.07 Temp	0.26
ε′	Without peel	2450	80.39 − 0.54 Met	0.98
ε″	Without peel	2450	19.48 − 0.20 Temp	0.88

Where, Met: methanol-water fraction (v/v), Temp: Temperature.

**Figure 4:**
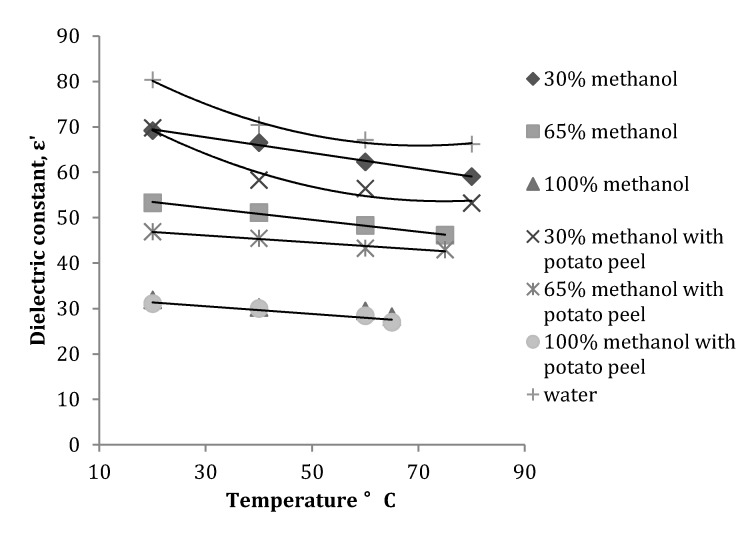
Variation in the dielectric constant of a methanol-water mixture with and without potato peel at 915 MHz (Source: Adapted from [9]).

**Figure 5:**
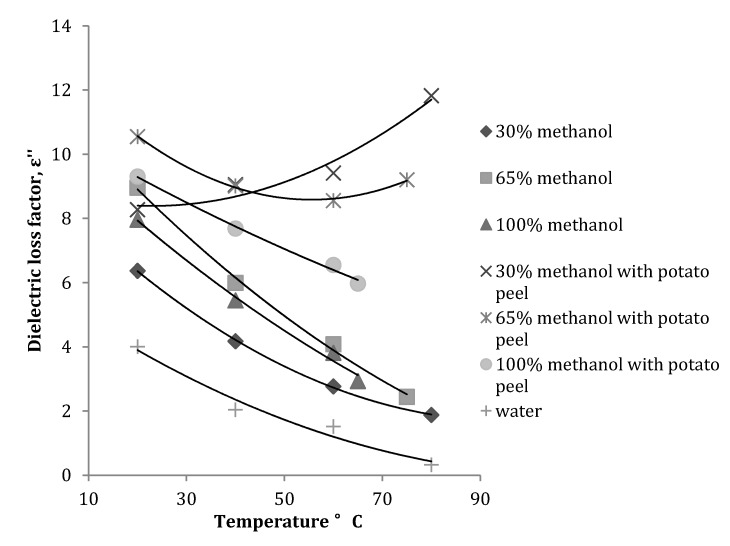
Variation in dielectric loss factor of a methanol-water mixture with and without potato peel at 915 MHz (Source: Adapted from [9]).

At 915 MHz, the dielectric constant of all the methanol fractions without potato peel decreased with an increase in temperature. With addition of potato peel into the solvent, the dielectric constant showed a similar trend, but the values measured were lower than the values measured without peel (Figure 4). The dielectric loss factor measured at 915 MHz showed a similar trend to that measured at 2450 MHz. For the 30% methanol fraction the dielectric loss factor gradually increased with increase in temperature (Figure 5). Dielectric loss factor values obtained for methanol fractions with peel were higher than the values without peel.

The regression analysis at 915 MHz revealed that the dielectric constant and dielectric loss factor were both significantly (*p* ≤ 0.05) affected by temperature rise and concentration of the methanol fraction. Introduction of the potato peel into the mixture had no effect on the significance of temperature and concentration on the dielectric constant, but compared to 2450 MHz, an increase in temperature had no significant (*p* ≥ 0.05) effect on the dielectric loss factor of the solvent mixture (Table 2).

**Table 2:** Equations defining the relationship between ε′ and ε″ with temperature and methanol fraction concentration at 915 MHz (Source: Adapted from [9]).

Dielectric Properties	Plant material	Frequency (MHz)	Equation obtained	R^2^
ε′	Potato peel	915	80.38 − 0.44 Met − 0.15 Temp	0.97
ε″	Potato peel	915	11.13 − 0.03 Met	0.36
ε′	Without peel	915	86.68 − 0.49 Met − 0.13 Temp	0.98
ε″	Without peel	915	8.60 + 0.012 Met − 0.096 Temp	0.9

Where, Met: methanol-water fraction (v/v), Temp: Temperature.

### 3.2. Effect of Temperature, Concentration and Presence of Plant Material on Dissipation Factor (tanδ)

**Figure 6:**
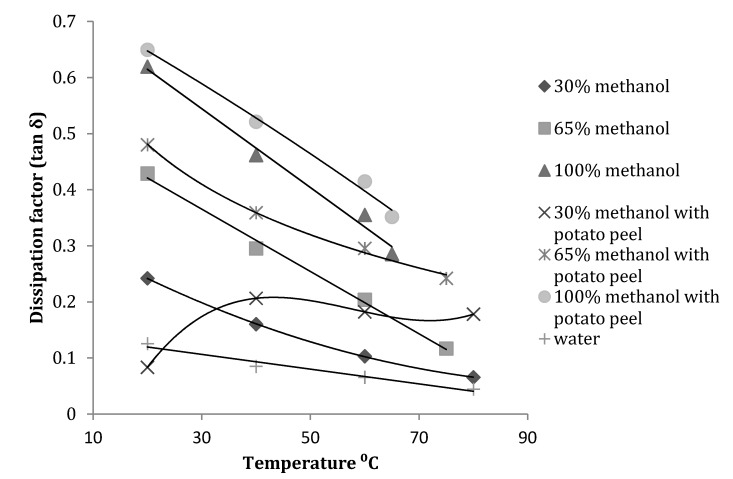
Dissipation factor of methanol-water mixture with and without potato peel at 2450 MHz (Source: Adapted from [9]).

It was observed that at 2450 MHz, the dissipation factor (tanδ) decreased with an increase in temperature for all methanol fractions. Even after introduction of potato peel the trend remained the same but the 30% methanol fraction had an increase in dissipation factor with introduction of the bio matrix (Figure 6).

**Figure 7:**
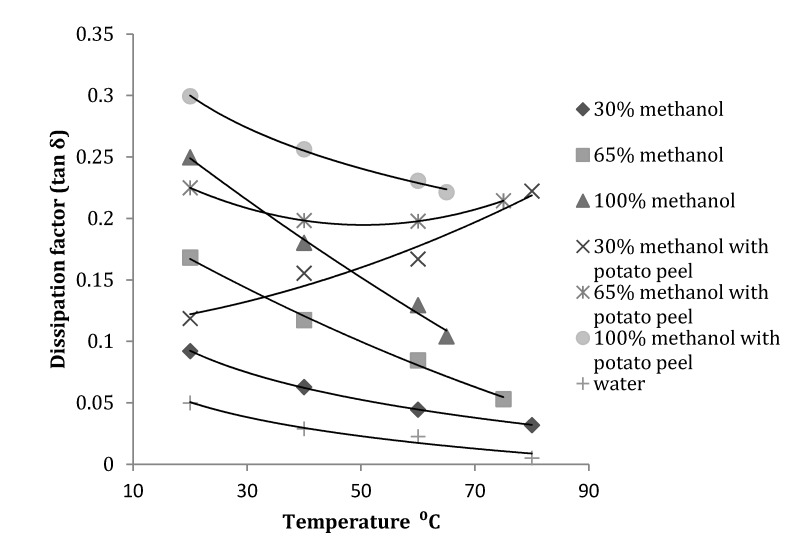
Dissipation factor of methanol-water mixture with and without potato peel at 915 MHz (Source: Adapted from [9]).

At 915 MHz, tanδ for the 30% methanol fraction with potato peel increased with an increase in temperature. For the 65% methanol fraction the value decreased with an increase in temperature until 40 °C and gradually increased until its boiling point of 75 °C. All other fractions showed a similar trend to that shown at 2450 MHz (Figure 7).

Statistical analysis of the data obtained for tanδ showed that both temperature and concentration had a significant (*p* ≤ 0.05) effect at 915 MHz and 2450 MHz. With introduction of potato peel at 915 MHz, tanδ was only affected by the change in the concentration of methanol and not by temperature (*p* > 0.05) and at 2450 MHz it was significantly affected by both temperature and the concentration of methanol in the mixture (Table 3).

**Table 3:** Equation obtained defining the relationship of dissipation factor (tanδ) with temperature and methanol fraction concentration at 2450 MHz and 915 MHz (Source: Adapted from [9]).

Dissipation factor	Plant material	Frequency (MHz)	Equation obtained	*R* ^2^
tanδ	Potato peel	915	0.12 + 0.00124 Met	0.60
tanδ	Without peel	915	0.11 + 0.00144 Met − 0.002 Temp	0.91
tanδ	Potato peel	2450	0.16 +0.005 Met − 0.003 Temp	0.79
tanδ	withoutpeel	2450	0.26 + 0.004 Met − 0.005 Temp	0.95

Met: Methanol-water fraction (v/v), Temp: Temperature.

### 3.3. Effect of Dielectric Properties of Methanol Fraction and Time on MAE Extraction of Antioxidant

**Figure 8:**
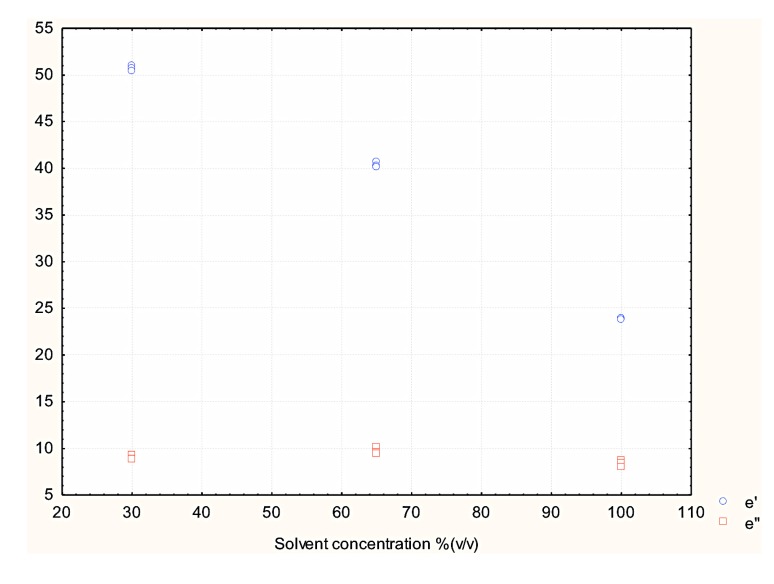
Effect of solvent concentration (v/v) on its dielectric constant and loss factor (Source: Adapted from [9]).

**Figure 9:**
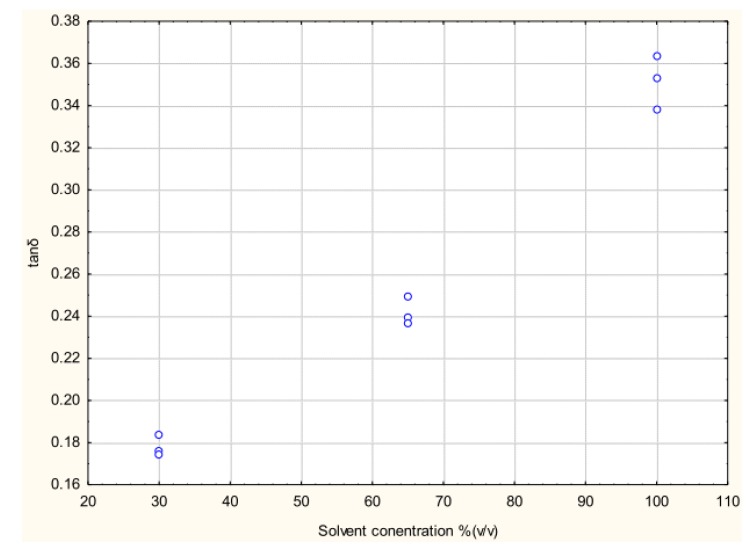
Effect of solvent concentration on dissipation factor (tanδ) (Source: Adapted from [9]).

**Figure 10:**
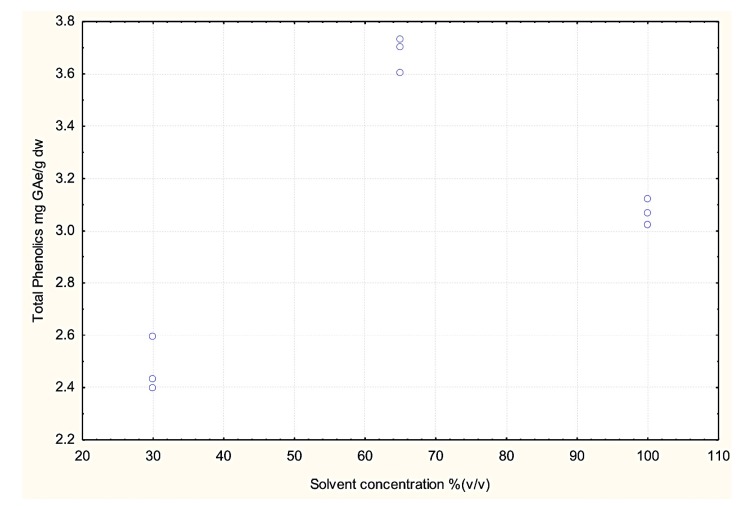
Effect of solvent concentration on total phenolic yield after extraction for 5, 10 and 15 min during the microwave-assisted extraction (MAE) process (Source: Adapted from [9]).

**Figure 11:**
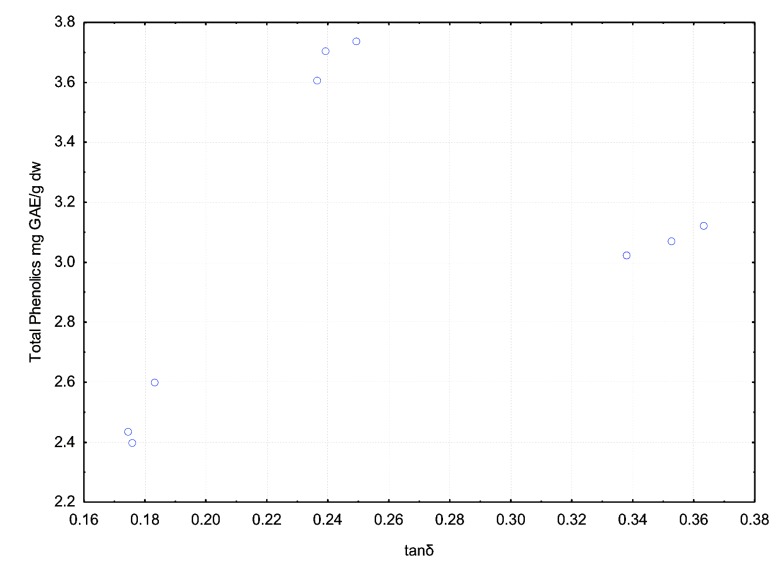
Effect of dissipation factor (tanδ) on total phenolic yield after extraction for 5, 10 and 15 min during the MAE process (Source: Adapted from [9]).

The success of the MAE process is determined by two major parameters, dielectric properties (ε′ and ε″) and time [11]. The dissipation factor (tanδ) determines the amount of heat dissipated during the process, it combines the effect of ε′ and ε″ on the amount of heat generated. In the present study the three methanol fractions were shown to have different dielectric properties and hence were expected to have a range of effects on microwave-assisted extraction of antioxidants from potato peel. The effects of the dielectric properties of the different methanol fractions used in the MAE extraction on the extraction yield of total phenolics of the potato peel extract are shown in Figure 8, Figure 9, Figure 10 and Figure 11.

The effects of the methanol concentration and its associated tanδ on total phenolics (TP) were significant (*p* ≤ 0.05; *R*^2^ = 0.70):

TP = 4.99 + (0.07 × Met) − (25.76 × tanδ) × *R*^2^= 0.70 (1)

Where, Met: Methanol-water fraction (v/v).

Figure 8 represents the effect of solvent concentration on ε′ and ε″. It was observed that ε′ decreased with increase in solvent concentration and ε″ remained largely unchanged. The effect of solvent concentration and tanδ on the total phenolic content of the potato peel extract can be observed in Figure 10 and Figure 11 respectively. The MAE extraction was conducted over different time periods (5, 10 and 15 min) and the highest total phenolic content was observed at the solvent concentration of 65% (v/v) at tanδ range of 0.23–0.25 and extraction time of 15 min.

Several researchers have stated that ε′ and ε″ are the dielectric properties which determine the type of solvent to be used in the MAE process [2,11,12,13,14,15,16]. From our study we suggest that along with ε′ and ε″, tanδ should also be considered when determining the suitable solvent, as it combines the direct effect of the dielectric constant and the dielectric loss factor on the MAE process.

Several researchers have suggested that higher water content in the solvent mixture has a negative effect on the extraction yield, because the higher water concentration increases the polarity to a degree where it is no longer favorable for the extraction process [10]. As observed in Figure 10, the highest total phenolic yield was obtained for the solvent concentration of 65% (v/v), *i.e.*, methanol-water ratio of 65:35. Similar results were obtained by Talebi *et al.* [17] during extraction of paclitaxel from *Taxus baccata*. They reported that a methanol-water (90:10) mixture gave the highest yield compared to mixtures with higher water content.

**Figure 12:**
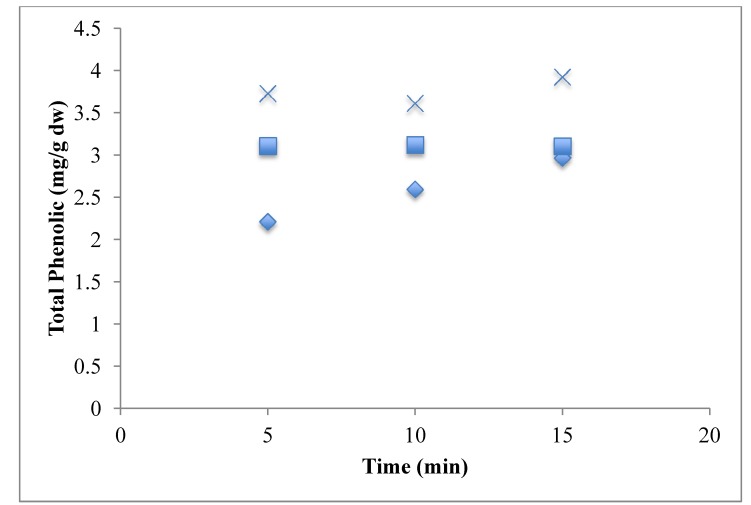
Effect of time and solvent concentration on total phenolic yield of the MAE process.

Note: ◆: 30% (v/v) methanol fraction, ■: 100% (v/v) methanol fraction and ×: 65% methanol fraction.

Time of extraction is also an important factor that determines the efficiency of the MAE process. The promotion of the MAE process is based on some of its advantages over conventional extraction methods, such as shorter extraction time and less solvent consumption. In our study on optimization of MAE for extraction of phenolic antioxidants from potato peel, we observed that time and solvent concentration play a significant role in determining the total phenolic yield [2]. Figure 12 represents the relation between time of extraction and solvent concentration on total phenolic yield from potato peel. It can be observed that the maximum yield was obtained after 15 min of extraction with a solvent concentration of 65% (v/v); the lowest yield was obtained for the 30% (v/v) solvent concentration. During the extraction process we also observed that the extraction temperature for the 30% (v/v) methanol fraction was around 85 °C compared to 70 °C and 65 °C for 65% and 100% methanol fractions respectively. Hence we can conclude that as the concentration of the methanol fraction was reduced, the extraction temperature increased, this is supported with the observations obtained for the 30% methanol fraction for which the dielectric loss and dissipation factors increased with an increase in temperature. Therefore as these values increased the temperature of the solvent during the extraction, more and more microwave energy was dissipated as heat and this led to a decrease in total phenolic yield due to loss of heat labile phenolic compounds.

## 4. Conclusions

Interaction of microwaves with the chemical components of a solvent and plant matrix can explain some of the phenomena behind the advantages of the MAE process. In this study we revealed that solvent concentration and temperature affect the dielectric properties of the solvent used during the microwave assisted extraction process. Understanding how the plant matrix interacts with the solvent under these changing conditions can explain how the extraction process is impacted. As per our analysis, the ε′ of the solvent fractions decreased with increase in temperature at both frequencies (915 and 2450 MHz) but when the plant matrix was introduced, the reduction rate was slow and the values were higher than the ones obtained for solvent fractions without plant material. Dissipation of the absorbed microwave occurs as the heat increases with the introduction of plant matrix in the solvent fraction. With respect to total phenolics extracted during the MAE process the yield is significantly affected by the solvent concentration, extraction time and dissipation factor of the solvent.
